# The Directional Observation of Highly Dynamic Membrane Tubule Formation Induced by Engulfed Liposomes

**DOI:** 10.1038/srep16559

**Published:** 2015-11-09

**Authors:** Xiaoming Zhang, Luru Dai, Anhe Wang, Christian Wölk, Bodo Dobner, Gerald Brezesinski, Yunqing Tang, Xianyou Wang, Junbai Li

**Affiliations:** 1 National Center for Nanoscience and Technology, 100190 Beijing, China; 2 National Lab for Molecular Sciences, CAS Key Lab of Colloid, Interface and Chemical Thermodynamics, Institute of Chemistry, Chinese Academy of Sciences, 100190 Beijing, China; 3 Institute of Pharmacy, Martin Luther University, 06120 Halle (Saale), Germany; 4 Max Planck Institute of Colloids and Interfaces, 14476 Potsdam, Germany; 5 Institute of Theoretical Physics, Chinese Academy of Sciences, 100190 Beijing, China

## Abstract

Highly dynamic tubular structures in cells are responsible for exchanges between organelles. Compared with bacterial invasion, the most affordable and least toxic lipids were found in this study to be gentle and safe exogenous stimuli for the triggering of membrane tubules. A specific lipid system was internalized by NIH3T3 cells. Following cellular uptake, the constructed liposomes traveled towards the nucleus in aggregations and were gradually distributed into moving vesicles and tubules in the cytosol. The triggered tubules proceeded, retreated or fluctuated along the cytoskeleton and were highly dynamic, moving quickly (up to several microns per second), and breaking and fusing frequently. These elongated tubules could also fuse with one another, giving rise to polygonal membrane networks. These lipid systems, with the novel property of accelerating intracellular transport, provide a new paradigm for investigating cellular dynamics.

Membrane-mediated transport is one of the major fields of focus in cell biology research. Membrane traffic between organelles is essential for a multitude of processes that maintain cell homeostasis. Membrane carriers are usually tubular- or vesicular-shaped structures. Typically, the tubular structures can be divided into two categories. The first class is structural tubules, which may play an important role in membrane architecture and function. Organelles such as the endoplasmic reticulum^1^,^2^, Golgi apparatus^3^,^4^, early endosome^5^, mitochondria^6^, tubular lysosomes^7^ and the intermediate compartment^8^ were found to be able to form tubular structures, and their movements and mechanisms of formation have been thoroughly investigated. The second class of tubules is highly dynamic and transient, and their movement is frequently driven by the cytoskeleton. Filopodia and other evaginations belong to this category. These tubules have been observed to emerge from the Golgi apparatus as well as from the endosomes and lysosomes and are believed to occur on all intracellular traffic routes. Moreover, cell biologists have discovered that the exchange of contents between late endosomes and lysosomes depends upon ongoing tubulovesicular late endocytic trafficking^9^, which is also responsible for exchanges between other organelles^10^.

In addition to these native filaments, it was recently been found that exogenous bacterial invasions^11^,^12^,^13^ can also induce highly dynamic tubular structures. Intracellular *Salmonella* could survive and replicate within a modified phagosome in HeLa cells. The onset of intracellular replication was accompanied by the appearance of membrane tubules, called *Salmonella*-induced filaments (Sifs), where SifA, a *Salmonella typhimurium* effector protein, was necessary for the formation of Sifs. Its C-terminal hexapeptide domain helped to anchor to the membrane, thus leading to fusion between the late endosomes and lysosomes.

However, not all bacteria (e.g., *E. coli*) can produce tubules in live cells^13^. During the processes of autophagy in immunity and cell autonomy, there is defense against intracellular microbes leading to their destruction. Moreover, bacterial invasion and the resultant infections are a risk factor for localized disease. So far, no candidates for the induction of highly dynamic membrane tubules by a gentle and safe exogenous stimulus have been found.

Most organelles are separated from their environment by a lipid membrane, and the shape and structure of these organelles are highly dependent on the membrane. The formation of membrane tubules has been shown to depend on the amount of cargo being transported^14^ and also requires specific proteins^15^. For example, NPC1-containing late endosomes can move by a dynamic process involving tabulation and fission, followed by rapid retrograde and anterograde migration along the microtubules^9^. COPII-coated vesicles form at the endoplasmic reticulum for cargo transport to the Golgi apparatus, where COPII proteins showed a preference for high curvature structures and induced beads-on-a-string-like constricted tubules^16^. SifA can help aid the formation of *Salmonella*-induced filaments (Sifs), as mentioned above^11^,^12^,^13^. Due to the highly dynamic relationship between proteins and lipids, associated proteins are likely to be concentrated within certain types of lipid domains^17^. Previous investigations suggested that the motors may interact directly with lipids in the membrane^18^, although the possibility has been raised that the motors may bind via integral membrane proteins. Compared with complicated protein modifications, membrane compositional changes may have important consequences on the localization of signaling proteins, which can in turn affect their activity.

Amino-functionalized and cationic lipids have offered various applications in the field of medicine. Constructed liposomes involving cationic lipids can be used as drug-delivery systems^19^, anti-microbial agents^20^, vaccine carriers^21^,^22^ and transfection agents^23^,^24^. Recent investigations have further demonstrated that lipid composition and liposome-entrapped agents have an impact on intracellular delivery^25^. Cationic lipids can usually activate cell signaling pathways, including pro-inflammatory and pro-apoptotic pathways^26^. New insights have also been made in the area of intracellular transport processes^27^.

In this study, a novel liposome involving lysine-based amino-functionalized lipid, N-{6-amino-1-[N-(9Z)-octadec-9-enylamino]-1-oxohexan-(2S)-2-yl}-N′-{2-[N,N-bis(2-aminoethyl)amino]ethyl}-2-[(9Z)-octadec-9-enyl]propandiamide (named OO4, the formula is shown in Fig. 1a) was described. The detailed synthetic process for this lipid was described in our previous publication^28^.

Compared to more common cationic lipids (e.g., DOEPC and DOTAP), the OO4 lipid increases the number of amino groups that can be protonated and, as a consequence, enlarges the head-group region. The lipid backbone consists of the proteinogenic amino acid lysine and a malonic acid unit. Its hydrophobic part contains two long oleyl chains. The first chain is bound via an amide bond to the lysine unit, and the second chain is bound to the α-carbon of the malonic acid unit, resulting in a larger distance between the alkyl chains in comparison to the alkyl chains in glycerol-based phospholipids. The use of lysine as a backbone and the high number of amide bonds determine the peptide-mimic character of the lipid. The head-group region was designed with free amino groups that can be easily protonated. Due to the biocompatibility and known complex formation ability of the proteinogenic amino acid lysine and DNA^29^,^30^, this structure may have specific polynucleotide binding properties and higher gene transfer efficiencies.

The produced liposomal complexes in NIH3T3 cells were fast-moving vesicles and tubules (up to several microns per second). They moved by a dynamic process involving tabulation and fission, followed by rapid retrograde and anterograde migration along microtubules. The moving speed was approximately five to ten times faster than that of normal transport vesicles in cells. In this regard, liposome invasion was like a catalyst that accelerated intracellular transport. The detailed structural properties for induced tubules were investigated by the direct stochastic optical reconstruction microscopy (dSTORM)^31^,^32^,^33^,^34^ technique. The motility and formation mechanism of the tubules were also studied in the following sections.

The results in this study provide a novel design idea for cellular dynamics in which liposome invasion can accelerate membrane-mediated transport. The most important finding is that the lipids used in this study are the most affordable and the least toxic. Compared with complicated protein modifications, functionalized lipids are expected to be an excellent candidate for accelerating intracellular transport and exchange.

## Results

### The internalization of liposomes

**Figure 1:**
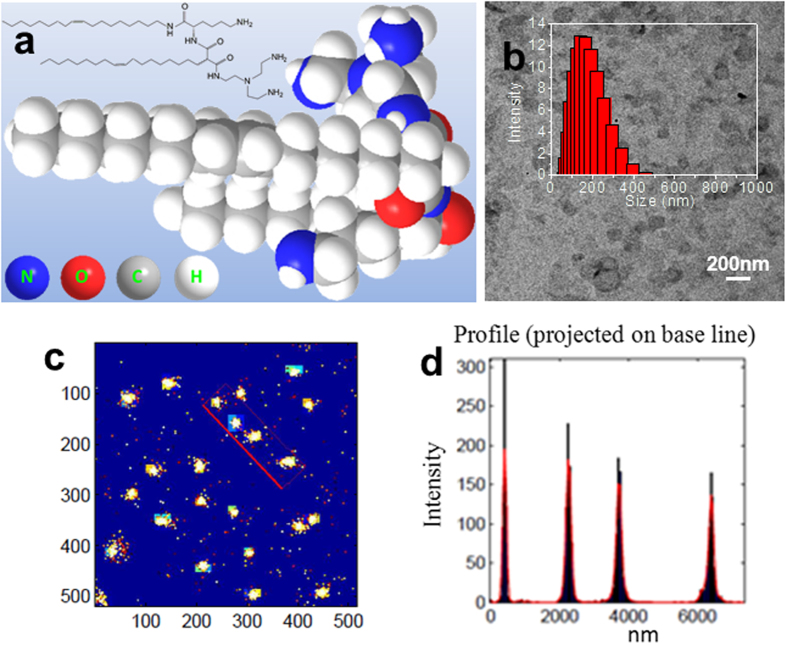
Structural characteristics of liposomes involving peptide-mimic lipids. (**a**) Chemical structure of lysine-based peptide-mimic cationic lipids. (**b**) TEM image for liposomes involving peptide-mimic lipids, and the inset shows DLS in aqueous solution. (**c**) Assembled liposome high resolution images in aqueous solution acquired by Gaussian fitting. The x and y axes are pixel numbers. (**d**) The profile for boxed area in (**b**) shows a 200–300 nm size distribution. Full-width-at-half-maximum (FWHM) value was measured by fitting the transverse profiles of localization with Gaussian. Column bar, 50 nm.

Liposomes were composed of the novel cationic lipid OO4 and the helper lipid dioleoylphosphatidylethanolamine (DOPE). They were prepared according to the standard protocol previously described. The TEM and DLS results suggested that these liposomes had an excellent distribution with a range of 150–250 nm, as shown in Fig. 1b. The ξ potentials of liposomes was approximately 36 mV. For this study, based on the knowledge that lysine is a main element of histones and poly-L-lysine has the ability to form complexes with DNA^25^,^27^, in addition to the abundant amine groups, we labeled the liposomes with Cy5-labeled oligonucleotide. The ξ potential measurements suggested that the liposomes were still positively charged after Cy5-labeled oligonucleotide coating (approximately 25 mV).

Through direct stochastic optical reconstruction microscopy (dSTORM), the localization of the marked liposomes in aqueous solution was imaged with nanometer precision. The images of liposomes using super resolution optical microscopy are achieved by the transition between a bright and a dark state of fluorescent dyes from liposomes, in order to assure that the fluorescence signal stems only from an isolated spot which is much smaller than the size of the diffraction limit. In this way, the object patterns can be resolved which are smaller than the diffraction limit. The measurement results revealed that these assemblies had excellent stability and a uniform size distribution at room temperature, as shown in Fig. 1c. The full-width-at-half-maximum (FWHM) value was analyzed by the fitting transverse profiles of localization with Gaussians (Fig. 1d), and the results showed a 200–300 nm size distribution that was consistent with the results from TEM and DLS.

**Figure 2:**
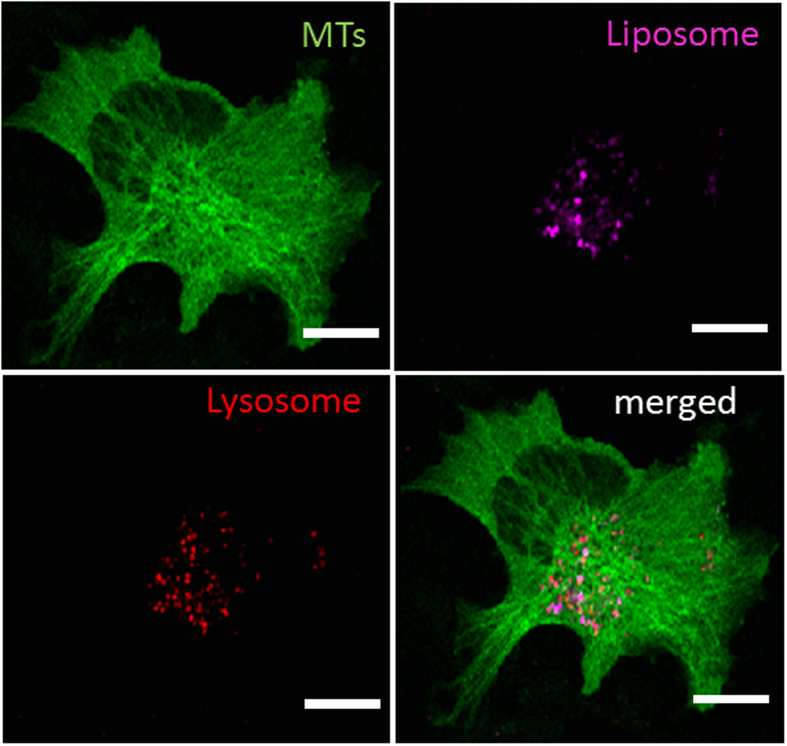
Confocal images of internalized liposomes in NIH3T3 cells. Green color shows microtubules (MTs) with GFP expression, magenta color shows liposomes loaded with Cy5-DNA, and red color shows lysosomes marked with Lyso-tracker red. The merged image suggests that the lysosomes overlaid most liposomes. Bar, 20 μm.

To explore the biological utility of liposomes, the marked liposomes were introduced into the media in which NIH3T3 cells were growing. Most of them could be internalized within 30 min at 37 °C. Following cellular uptake, the liposomes traveled towards the nucleus (3–6 h) in aggregations and gradually distributed into moving vesicles and tubules in the cytosol (over 12 h). To confirm the intracellular location, the viral vector Cell Light Tubulin-GFP was transfected into the cells to mark the microtubules. Most liposomes were found to distribute in the area where numerous MTs existed, as shown in Fig. 2. Previous studies demonstrated that endosomes and lysosomes were usually involved after exogenous invasion^11^,^12^,^13^. Such a co-localization phenomenon could be validated by LysoTracker labeling. As expected, most aggregates were associated with LysoTracker-positive lysosomes (see the merged confocal image in Fig. 2).

### Configuration changes of internalized liposomes

**Figure 3:**
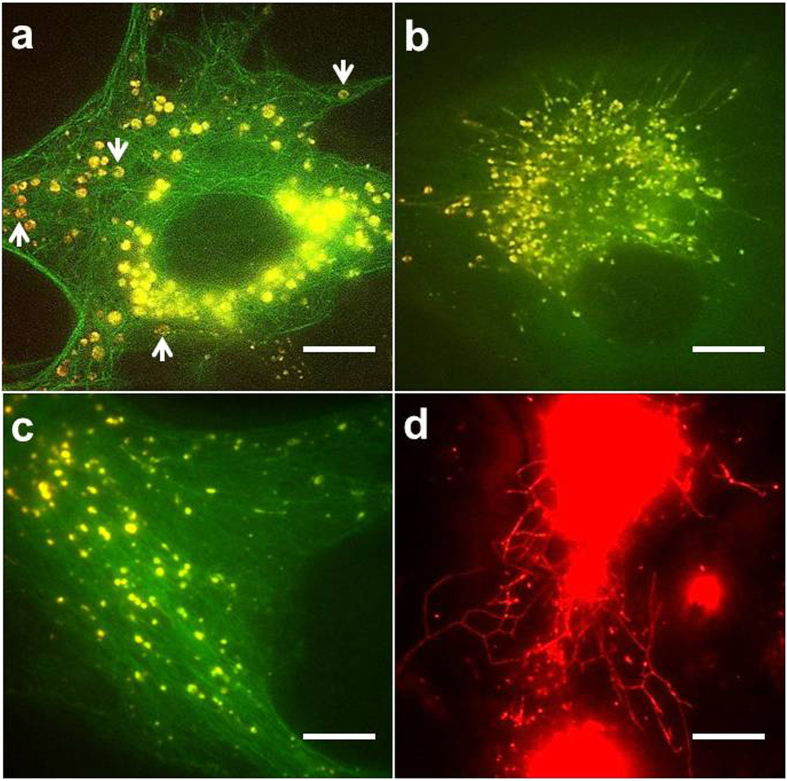
Representative wide-field images of the tubular network formation process in NIH3T3 cells. (**a**) Large and globular domains are formed at an early stage (incubation within 3 h). At this stage, most domains are relatively stationary and in loop-like structures, shown by white arrows. (**b**) Membrane tubules begin to appear from domain extensions along the microtubules (MTs) at a later time (6–12 h). (**c**) Plenty of actively tubular and vesicular liposomal complexes subsequently appeared. (**d**) Formation of polygonal membrane networks involve the movement of membrane tubules. Notes: Green color (**a**–**c**) shows GFP-expressed MTs, and yellow color shows internalized liposomes, which are labeled by Cy5. To exclude the effects from expressed proteins, no transfection was applied to the cell in (**d**), thus, the red color was entirely generated by the internalized liposomes. Bar, 10 μm.

According to wide-field microscopic images (Fig. 3), these aggregates exhibited a constantly changing morphology over time. Once internalized by the cell membrane, these liposomes preferred to gather to some circular domains in a restricted manner, where the liposomes interconnected and formed a tight ‘matrix’. Interestingly, these matrices themselves were not uniform but were mostly located along the rim of the circle. Most liposomes presented Brownian motion within a restricted area surrounded by the cytoskeleton, as shown in Fig. 3a and Supplementary Movie 1. Later, moving vesicles began to originate from these domains, and membrane tubules began to form and elongate by moving along a microtubule track (Fig. 3b and Supplementary Figure S1). With increasing time, these complexes involving tubules and vesicle-like domains were distributed towards the periphery of the cell. During this process, the formative tubules proceeded, retreated or fluctuated along the MTs with a stop-and-go running, and sometimes, some short tubular structures were observed to connect at their tips to form one continuous long tubule, or one long tubule detached into two or more independent short tubules or single vesicles (as shown in Fig. 3c and Supplementary Movie 2). This observed phenomenon was consistent with the results of previous studies, in which similar tubule structures induced by bacteria were observed^11^,^12^,^13^. More interestingly, these tubules can also reversibly form circular structures, where only one site is anchored to MTs, while the other parts are free in the cytosol.

The most exciting result was that an extensively branched network could be induced by these exogenous liposomes, with abundant tubules extending from aggregates and proceeding and retreating at very fast rates (Fig. 3d and Supplementary Movie 3). It should be noted that to exclude the potential effects from expressed GFP proteins and organic dye molecules, no tubulin-transfected or Lyso-Tracker agents were applied here, and therefore, the signals (marked in red color) in Fig. 3d arose only from the liposomes. The tubules continuously changed size, detached from junctions, and rejoined to form new tubules. These reticular structures themselves were highly dynamic at longer timescales.

### The dynamic behaviors of internalized liposomes

We also found that the dynamic behavior strongly depended on the morphology of the internalized liposomes (see Supplementary Figure S2). For optimal sensitivity and speed, high-speed wide-field microscopy was utilized to conduct the optical measurement of the (x, y) position of the single object as a function of time. Most of the re-dispersed liposomal complexes were relatively free and conduct Brownian movement in a restricted area. They extended into short tubules or moved along MTs only when they anchored to MTs and were dragged by suitable force from motor proteins. Note that the subsequent analysis only used long trajectories of more than 1 μm to measure the transportation speed with reasonable accuracy. Thus, only the actively directed transport velocities of individual liposomes and induced tubules were analyzed. Their highly dynamic movement was tracked and precisely mapped, and calculations were also conducted (Supplementary Figure S3).

**Figure 4:**
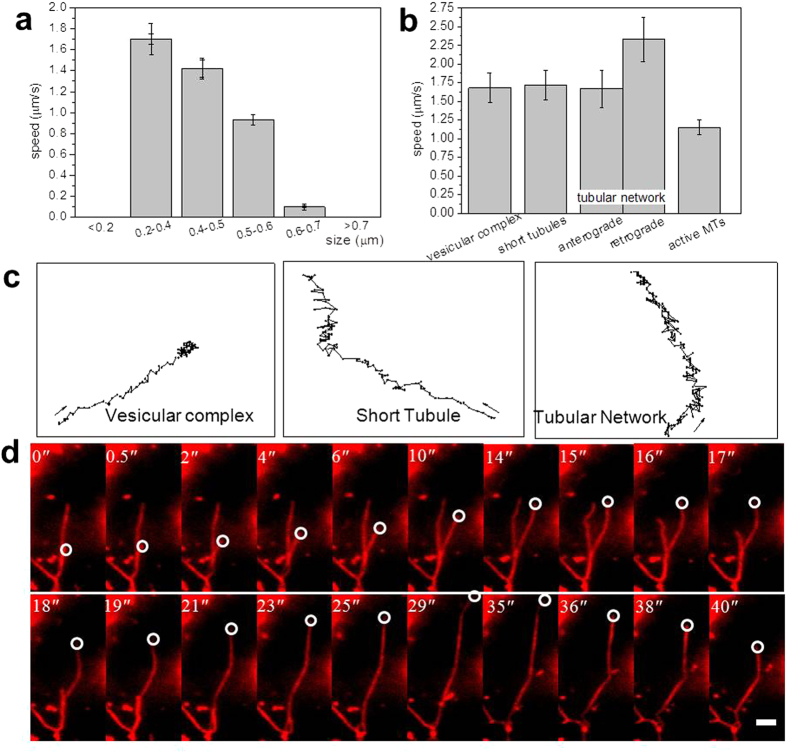
Dynamic characteristics of membrane compartment motility. (**a**,**b**) Histogram showing the average velocities of these compartments with different diameters and trafficking patterns, respectively. The error bar is ±SD. (**c**) The tracking maps of a single vesicular complex, short tubules and the tubular network demonstrate that these membrane structures fluctuate bi-directionally, but with an overall bias in one direction. The tracks were followed in the same timescale. The start point was shown by arrows. (**d**) The time lapse images show that the tubules (only tips was marked) can extend and cross, branch and retract, giving rise to a 3-way junction with very high motility. Bar, 2 μm.

Most mono-disperse liposomes should be integrated into complexes involving lysosomes, as demonstrated in the section “The internalization of liposomes.” The evidence for co-localization between lysosomes and liposomes indicates that the liposomes were associated or fused with lysosomes. Interaction or content exchange between liposomes with cellular membrane lipids and membrane organelle lipids (such as lysosomes) may result in a different organization compared to the original structure. The statistics suggested that the speed of these liposomal complexes was proportional to their size, as shown in Fig. 4a; the fast-moving vesicles were relatively small in diameter, ranging from 0.2 μm to 0.7 μm. Vesicles larger in size than 0.7 μm (which were, in fact, vesicle aggregations) were excluded because they remained relatively stationary and conducted Brownian movement in a restricted area most of the time. However, interestingly, free and short tubules (usually several micrometers length) had a mean velocity similar to that of smaller vesicles, as shown by the statistical results of different tracking patterns in Fig. 4b, which suggested that their speed was not exclusively related with mass. Perhaps steric hindrance played an important role in the larger liposomal complexes due to the complicated intracellular environment. It should be noted that the statistics for the mean transport velocity were derived from at least 20 targets for each tracking pattern in different cells and different experimental batches.

The dynamic behavior also strongly depended on MT density (Supplementary Figure S4). According to the statistical results in Fig. 4b, we found that the velocities (>1.4 μm/s, average ± SD) of vesicular complexes were much greater than those of native lysosomes (0.44 μm/s) and autolysosomes (0.33 ± 0.04 μm/s and 0.39 ± 0.05 μm/s for anterograde and retrograde transport, respectively) within the cytosol^35^,^36^. Most liposomal complexes moved or fluctuated steadily along MTs at long timescales; over this period, they proceeded, retracted, occasionally touched or agglutinated with others. In some cases, two liposomal complexes could be seen moving along the same fiber in opposing directions and passing each other without colliding. Sometimes, they would suddenly change their velocities and move quickly by proceeding or retracting; the maximal speeds were 3.99 μm/s and 5.24 μm/s, respectively. Usually, MTs were relatively stationary with only minor fluctuations in the vertical and elongation directions. However, for active MTs, they could sometimes produce a much higher retraction speed (Fig. 4b and Supplementary Movie 4). Furthermore, there was significant evidence that cargoes were transported *in vivo* by multiple motors^37^,^38^. Therefore, the sudden enhancement of transport may involve the cooperation of MTs, numerous motor proteins, and the changing of cytosolic microenvironments as well. A similar phenomenon was also observed by Y. Yang *et al*., for which the speed in the retrograde direction was also higher than that of the anterograde direction for autolysosomes under starvation conditions^35^.

For the tubule network, they moved steadily with tips forward or backward. They moved very quickly (with a peak instantaneous velocity at 4.69 μm/s, and 5.40 μm/s for anterograde and retrograde transport, respectively), suggestive of elastic recoil. Moreover, retrograde transport was always faster than anterograde transport. The experimental results demonstrated that the establishment of tubular networks involved an increase in dynamics and stability. The overlay images of trajectories mapped by 2D Gaussian fitting demonstrated that these reticular structures could maintain a higher stability at a long timescale and consistently move either anterograde or retrograde with few changes in direction (Supplementary Figure S5). This was consistent with the conclusion from Fournier *et al*., where they found that tubular shapes could form the most stable state by taking into account fourth-order membrane elastic theories^39^.

Interestingly, different morphological complexes exhibited similar tracking behaviors. The spatial positions of the moving targets were determined, and consecutive positions were connected and shown as a tracking map. The tracks of vesicular, tubular and reticular complexes within 10 s are shown in Fig. 4c, on the same axis scales. These tracks suggested that these membrane compartments switched frequently between periods of extension, retraction and oscillation towards certain directions. A series of single optical planes taken from one time-lapsed series revealed that tubule growth or extension was bidirectional and involved switching between elongation and retraction (Fig. 4d). The bidirectional nature of the movement suggested the involvement of distinct minus-end-directed and plus-end-directed motors.

### The possible reasons for tubule formation

To ensure that the observed structures were not a universal phenomenon, a number of control experiments were performed. Liposomes (DOPE:DMPA:DHPE-RhB 75%:24%:1%) were added to culture medium for the same incubation time, and similar but shorter tubules were observed. However, there was no obvious aggregation or tubule formation for the zwitterionic liposome system (DOPE:DMPC:DHPE-RhB 75%:24%:1%). In addition, no obvious tubules were observed when only Cy5-DNA oligo or cationic dipeptide particles (no lipids were involved to exclude the effect from pure positive charges) were internalized by 3T3 cells. These results indicated that the observed morphologies indeed depended on the lipid compositions. Although DOPE-containing complexes showed great variability in the morphology of the complexes^40^, the role of DOPE could be neglected based on the control experiments. The biggest difference between the three liposomal systems was the head-group region of working lipids. The novel lysine-based lipid was positively charged under physiological conditions due to abundant primary amino groups that could be easily protonated. DMPA is a negatively charged lipid, while DMPC is neutral. Thus, we arrived at the hypothesis that the electric charges or head group structures of lipids played an important role in tubule formation. However, cationic liposomes constructed with DOTAP:DOPE (1:3), the most popular transfection agent, were used as a control. As expected, no tubules were observed. This result suggested that a positive charge was not vital for tubule formation. In addition, thus far, no similar phenomenon has been observed by other scientists in other cationic lipid systems such as popular liposomal drug carriers or transfection agents.

The only explanation for this activity is the unique structure of lipids. It has been reported that lipids with polyunsaturated hydrocarbon chains facilitate membrane fusion^41^, which was explained by the surprising flexibility of polyunsaturated hydrocarbon chains that allowed for large changes in effective chain length without energy penalties, therefore making it easier to fill the so-called packing voids of fusion intermediates. Here, in comparison to the alkyl chains in glycerol-based phospholipids, a larger distance between the alkyl chains made them more flexible. Another property was the abundance of involved amine groups compared with common cationic lipids such as MDTAP and DOTAP, where only one amine is involved.

Taken together, based on the highly dynamic relationship between proteins and lipids, as well as their unique properties, we can assume that in addition to the membrane instability induced by the lipid structure itself, this unique lipid may contribute to the restriction and assembly of proteins (such as kinesin, dynein *etc.*) or special receptors to particular regions of the membrane compartments, which facilitate the formation of a continuous membrane structure. It is also conceivable that the clustering of motors and correlative proteins into domains generates a force that could aid in the budding process in some situations, thus leading to small liposomal complexes or short tubules detaching from and rejoining their parent structures.

### Structural characteristics of liposomal complexes and induced tubules

**Figure 5:**
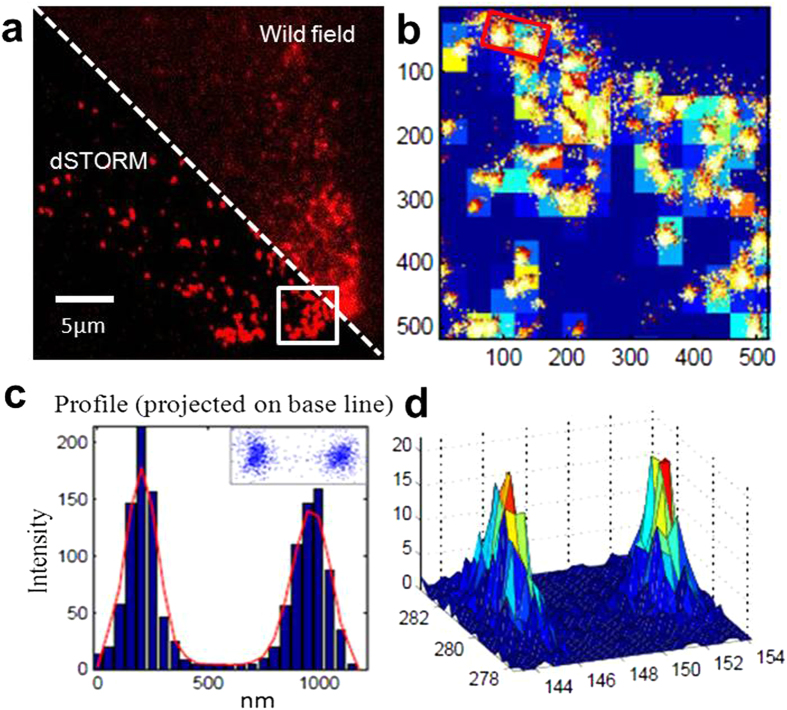
Internalized vesicular liposomal complexes in NIH 3T3 cells. (**a**) Wide-field image of liposomal complexes engulfed by NIH3T3 cell is shown on the right top, and a dSTORM high resolution image is shown on the left bottom. (**b**) Gaussian fitting image for the regions of interest (box in a). (**c**) The box area in (**b**), binned with 50 nm intervals, was then fitted with Gaussians to determine the width, as indicated in the plots. The insert is the corresponding dot distribution. (**d**) The corresponding 3D-view profile for the boxed area. The x and y axes are pixel numbers in (**b,d**).

For mono-dispersed liposomal complexes, nanometer-precision localization was achieved by fitting a Gaussian distribution function to the intensity of the observed fluorescence spot. Higher irradiation intensity was applied to transfer the majority of Cy5 dyes into a non-fluorescent OFF state until a sufficiently low spot density for each object was reached. Finally, a well-resolved dSTORM image was reconstructed from thousands of frames. Individual object images with higher signal-to-noise ratios were obtained compared to wide-field images, as shown in Fig. 5a. A Gaussian fitting image for the regions of interest showed that these liposomal complexes were independently distributed (Fig. 5b). The full-width-at-half-maximum (FWHM) values (Fig. 5b) and corresponding Gaussian fitting dot distribution (Fig. 5d) demonstrated that there were no clear changes in size after liposomal endocytosis. From a thermodynamic point of view, liposomes or analogues are not at equilibrium but represent kinetically trapped systems. According to the basic DLVO (Derjaguin–Landau–Verwey–Overbeek) theory^42^, the abundant amino groups on the surrounding surface cause an electrostatic repulsion between neighboring liposomal complexes that is larger than their van der Waals attraction. The interplay between these two types of opposing forces makes liposomal complexes stable at physiologically relevant conditions. It should be noted that interaction or content exchange between liposomes with cellular membranes or organelle lipids may result in a different organization compared to the original structure, which may lower the number of water binding sites or charges on the membranes, thus decreasing lipid bilayer repulsion and facilitating the close contact of the membranes.

**Figure 6:**
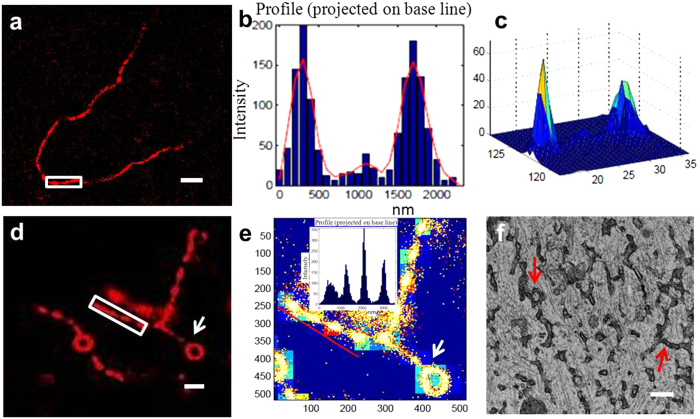
Detailed structures of tubular compartments in NIH3T3 cells. (**a**) dSTORM imaging of typical tubular structures. Both the 2D profile (**b**) and the 3D view (**c**) of the box area suggest a discontinuous linear array of liposomes. Column bar is 100 nm. (**d**) Typical high resolution structures with tubules and loops. (**e**) The inserted transverse profiles of the box areas also show a neck-like structure, while in the ring part (pointed by a white arrow), the tubules remain continuous. Column bar is 50 nm. (**f**) Similar tubular and loop structures also appeared in the TEM image. Scale bar, 500 nm.

To investigate the details of tubular structures, the cells were immediately fixed after dynamics observation. Unfortunately, not all tubules were properly preserved during fixation, which suggested that some related proteins were involved in the tubule formation process. A representative tubular structure was investigated via super-resolution imaging of discontinuous linear arrays of liposomes, also referred to as ‘beads-on-a-string,’ interspersed with tubular profiles (Fig. 6a). Nanometer-sized structural kinks in the seemingly continuous lines were unveiled. Both the 2D profile (Fig. 6b) and the corresponding fluorescence signal distributions (Fig. 6c) suggested a discontinuous linear array of liposomes.

It is well known that the tubular network of the endoplasmic reticulum (ER) is generated and maintained by a class of membrane proteins, where these proteins partition into and stabilize highly curved ER membrane tubules^43^. Here, the damaged proteins that resulted from cell fixing could no longer support the tubular structure, especially for longer tubules and reticular structures. Thus, many neck-like structures appeared along the tubule track and separated the tubules into pearl-like structures. Similar phenomena were also observed in some other tubular structures with rings (as shown in Fig. 6d). Interestingly, in the ring parts, the tubules were maintained as continuous. While in the strip part, the structure became discontinuous (Fig. 6e). These results perhaps predicted the differences in protein (such as motor proteins) function and cooperation on bent and strip orbits. Electron microscopy results also supported our hypothesis, as shown in Fig. 6f; vesicles and quasi-tubules were arrayed along clear MTs rails where junction and circular structures were also observed (noted by arrows). The size gap of measurements of liposomal complexes might be due to the differences between the hydrated state in fluorescence imaging and the dried state in TEM ultrathin sections.

## Discussion

In this study, a specific lipid system involving a peptide-mimetic lipid with a high number of amide bonds and a lysine backbone was strongly internalized in NIH3T3 cells. For the first time, the introduction of extensive tubular compartments into epithelial cells by liposomes or lipid invasion was experimentally observed. The induced tubular compartments moved along MTs in both the anterograde and retrograde directions. Their movement involved frequent stops, re-starts and direction switching. Some of them shared exactly the same transport pathway. This dynamic phenomena was very similar to most other tubular endomembrane systems in common mammalian cells such as the ER and tubular lysosomes. However, in contrast to these structural tubules, the tubules involving peptide-mimetic cationic lipids were highly dynamic and presented extreme, long-term high stability. Our experimental data provides new insight into liposome-induced tubular architecture and its structural dynamics in intracellular transport.

Due to the markedly acidic environment within lysosomes, once abundant protonable amino groups associate or fuse with lysosomes, they may recruit and anchor motor proteins or other correlative ligands to the membrane, thereby altering their conformation and enhancing catalytic activity. Alternatively, instead of modifying the proteins, membrane composition changes may take place at the membrane organelles via lipid invasion and exchange, which can have important consequences on the localization of signaling proteins which can in turn affect their activity. There is clear evidence that a system’s performance is strongly affected by the way the motors are linked to the cargo^36^. It is also possible that a designed lipid allows membranes to form an impressive variety of shapes to divide, migrate, communicate with other cells, initiate organelle biogenesis, and enable trafficking.

## Materials and Methods

### Lipid synthesis and liposome preparation

The lipid OO4 was synthesized (see Supplementary method) using preparation procedures previously described^28^. 1,2-dipalmitoyl-sn-glycero-3-phosphoethanolamine-N-lissamine rhodamine B sulfonyl (DHPE-RhB), 1,2-dimyristoyl-sn-glycero-3-phosphocholine (DMPC), 1,2-dioleoyl-sn-glycero-3-phosphoethanolamine (DOPE), and 1,2-dioleoyl-3-trimethylammonium-propane (chloride salt) (DOTAP) were purchased from Avanti Polar Lipids, Inc. The mixed lipids (DOPE:OO4, 3:1, mol/mol) were dissolved in chloroform in a round bottom flask. The chloroform was evaporated by a rotary evaporator, and distilled water was added to a final concentration of 0.5 mg/mL. Liposomes were prepared through two 200-nm polycarbonate filters using a mini-extruder from Avanti Polar Lipids. After extrusion, the lipids were stored at 4 °C before use.

### Cell culture

The NIH3T3 cell line was grown in Dulbecco’s modified Eagle’s medium (DMEM, GIBCO-BRL, New York, USA) supplemented with 10% fetal calf serum (FBS, Biochrom, Berlin, Germany) at 37 °C in a humidified environment containing 5% CO_2_. To view the MT skeletons, the cells were transfected via viral vector transduction (Cell Light Tubulin-GFP). Invasion with an appropriate amount of liposomes was carried out one day after preparation. To exclude the effect from serum proteins, incubation involving liposomes for 1 hr in Hank’s balanced salt solution (HBSS) was performed, and then, the medium was immediately changed back to DMEM. Before imaging, cells were washed twice with HBSS and kept in HBSS for observation by fluorescence microscopy.

### Cell imaging acquisition

Cells were visualized by an inverted microscope (IX-81, Olympus) with a 150 × 1.45 NA oil immersion objective (PlanApoN, Olympus) using standard filter sets and lasers. Sequential images were captured with a cooled charge-coupled device (CCD) camera (iXon, ANDOR Technology); the image resolution was 106 nm/pixel. For two-color imaging, excitation and emission light were separated via a multiband dichroic mirror (Di01-R405/488/561/635-25 × 36 quad-edge laser flat dichroic beam splitter) in combination with a multiple bandpass filter (FF01-446/523/600/677-25 quad-band bandpass filter). The mapping speed was dependent on the ROI (region of interest). Both channels were recorded by an EMCCD camera running at 10 Hz with 512 × 512 pixels unless it was specifically stated otherwise. The temperature was maintained at room temperature. The velocities were quantified frame to frame using the ImageJ macro manual tracking.

For the fixed cell imaging experiments, except for the oxygen scavenging system based on the addition of glucose oxidase and catalase, the reducing agent β-mercaptoethylamine at a final concentration of 100 mM was also necessary to induce photoswitching between a fluorescent on-state and a stable non-fluorescent off-state. The images of vesicles using super resolution optical microscopy are achieved by the transition between a bright and a dark state of fluorescent dyes from vesicles, in order to assure that the fluorescence signal stems only from an isolated spot which is much smaller than the size of the diffraction limit. In this way, the object patterns can be resolved which are smaller than the diffraction limit. Making full use of photoswitchable probes (Cy5) to switch reversibly between the on and off state in this study, a higher irradiation intensity was applied to transfer the majority of Cy5 dyes into a non-fluorescent OFF state to assure that the fluorescence signal stemmed only from an isolated spot much smaller than the size of the diffraction limit. This way, object patterns could be resolved which are smaller than the diffraction limit. Finally, a well-resolved dSTORM image was achieved by fitting a Gaussian distribution function to the intensity of the observed fluorescence spots (obtained via the SNSMIL method^44^) from thousands of frames. All analyses were carried out using a custom-written MATLAB code. The regions of interest were selected with boxes, and the histograms were fitted with Gaussians and FWHM values binned with intervals as indicated in the plots.

For transmission electron microscopy imaging, NIH 3T3 cells were fixed with 2.5% glutaraldehyde in 0.1 mol/L PBS (pH 7.4) solution, and then ultrathin sections (70 nm) were created using a standard protocol. The thin sections were stained with uranyl acetate and lead citrate and then visualized with a transmission electron microscope operating at 100 kV.

## Additional Information

**How to cite this article**: Zhang, X. *et al*. The Directional Observation of Highly Dynamic Membrane Tubule Formation Induced by Engulfed Liposomes. *Sci. Rep.* **5**, 16559; doi: 10.1038/srep16559 (2015).

## Supplementary Material

Supplementary Information

Supplementary Video 1

Supplementary Video 2

Supplementary Video 3

Supplementary Video 4
