# Supplementary material for: The Directional Observation of Highly Dynamic Membrane Tubule Formation Induced by Engulfed Liposomes
Source: Sci Rep. 2015 Nov 9;5:16559. doi: 10.1038/srep16559 (PMC4637876; doi:10.1038/srep16559)
Supplement: Supplementary Information [file srep16559-s1.doc]

Supporting information for

**The Directional Observation of Highly Dynamic Membrane Tubules Formation Induced by Engulfed Liposomes**

Xiaoming Zhang^1, 2^, Luru Dai^1^, Anhe Wang^1^, Christian Wölk^3^, Bodo Dobner^3^,Gerald Brezesinski^4^, Yunqing Tang^1,5^, Xianyou Wang^1,5^and Junbai Li^1,2,*^

^1^ National Center for Nanoscience and Technology, 100190 Beijing, China;

^2^ National Lab for Molecular Sciences, CAS Key Lab of Colloid, Interface and Chemical Thermodynamics, Institute of Chemistry, Chinese Academy of Sciences, 100190 Beijing, China;

^3^Institute of Pharmacy, Martin Luther University, 06120 Halle (Saale), Germany;

^4^Max Planck Institute of Colloids and Interfaces, 14476 Potsdam, Germany;

^5^ Institute of Theoretical Physics, Chinese Academy of Sciences, 100190 Beijing, China;

^*^To whom correspondence should be addressed. Email:　[jbli@iccas.ac.cn](mailto:jbli@iccas.ac.cn).

**This file includes:**

Lipid synthesis method;

Figure S1 to S5;

Movie S1 to S4

Su**pplementary method (OO4 synthesis):**

Scheme of Synthesis (DIPEA = diisopropyl ethyl amine, TEA = triethyl amine, TFA = trifluoro acetic acid, THF = tetrahydrofuran):

(2*S*)-2-Amino-6-[(*tert*.-butoxycarbonyl)]amino-*N*-[(9*Z*)-octadec-9-enyl]hexanamide(**compound A**), which was described in our previous paper by Wölk et al (*Chem. Eur. J.* **19**, 12824-12838(2013)).

(11*Z*)-2-[(*N*-{6-[(*tert*.-Butoxycarbonyl)amino]-1-{*N*-[(9*Z*)-octadec-9-enyl]amino}-1-oxohexan-(2*S*)-2-yl}amino)carbonyl]icosa-11-enoic acid ethyl ester (**Compound B**) M(C_52_H_97_N_3_O_6_) = 860.34 g mol^-1^

Yield: 97 %; melting point: 55-58 °C; R_f_: 0.50 (CHCl_3_/ethyl ether, 1/1, v/v); ESI-MS: 882.6 [M+Na]^+^; elementary analysis: calc. C=72.59 H=11.36 N=4.88, found C=72.48 H=11.27 N=4.85; ^1^H-NMR (CDCl_3_, 500 MHz, 27 °C) δ[ppm]= 0.88 [t, ^3^J(H,H) = 6.8 Hz, 6H, 2×‑(CH_2_)_2_CH_3_], 1.26-1.93 [m, 68H, -CH_2_-alkyl, -(CH_2_)_3_CH_2_NHBOC, -OCH_2_CH_3_, -OC(CH_3_)_3_], 1.93-2.05 [m, 8H, 2×-CH_2_CH=CHCH_2_-], 3.05-3.24 [m, 5H, -CH_2_NHBOC, -CH_2_NHCO-, -COCH(oleyl)CO-], 4.16-4.23 [m, 2H, -OCH_2_CH_3_], 4.31-4.36 [m, 1H, -COCH(NHCO-)CH_2_-], 4.60 [s, 1H, -NHBOC], 5.31-5.40 [m, 4H, 2×-CH=CH-], 6.19/6.31 [2×s, 1H, -CH_2_NHCO-], 6.90-6.95 [m, 1H, -COCH(NHCO-)CH_2_-]

(11*Z*)-2-[(*N*-{6-[(*tert*.-Butoxycarbonyl)amino]-1-{*N*-[(9*Z*)-octadec-9-enyl]amino}-1-oxohexan-(2*S*)-2-yl}amino)carbonyl]icosa-11-enoic acid (**compound C**) M(C_50_H_93_N_3_O_6_) = 832.29 g mol^-1^

Yield: 97 %; melting point: 59-60 °C; R_f_: 0.39 (CHCl_3_/methanol, 9/1, v/v); ESI-MS: 830.4 [M-H]^-^; elementary analysis: calc. C=72.15 H=11.26 N=5.05, found C=71.94 H=10.91 N=5.13; ^1^H-NMR (CDCl_3_, 500 MHz, 27 °C): δ[ppm]= 0.86 [t, ^3^J(H,H) = 6.9 Hz, 6H, 2×‑(CH_2_)_2_CH_3_], 1.26-1.86 [m, 65H, -CH_2_-Alkyl, -(CH_2_)_3_CH_2_NHBOC, -OC(CH_3_)_3_], 1.94-1.99 [m, 8H, 2×-CH_2_CH=CHCH_2_-], 3.06-3.24 [m, 5H, -CH_2_NHBOC, -CH_2_NHCO-, -COCH(oleyl)CO-], 4.40-4.46 [m, 1H, -COCH(NHCO-)CH_2_-], 4.81 [s, 1H, -NHBOC], 5.30-5.36 [m, 4H, 2×-CH=CH-], 6.95-7.15 [m, 1H, -NHCO-], 7.41-7.62 [m, 1H; -NHCO-]

*N*-{2-[*N*,*N*-Bis(2-aminoethyl)amino]ethyl}-*N*`-[6-[*N*-(*tert*.-butoxycarbonyl)amino]-1-{*N*-[(9*Z*)-octadec-9-enyl]amino}-1-oxohexan-(2*S*)-2-yl]-2-[(9*Z*)-octadec-9-enyl]propandiamide (**compound D**) M(C_56_H_109_N_7_O_5_) = 960.51 g mol^-1^

Yield: 94 %; melting point: 74-80 °C; R_f_: 0.28 (CHCl_3_/methanol/NH_3_, 80/20/2, v/v/v); ESI-MS: 961.6 [M+H]^+^; elementary analysis: calc. C=68.73 H=11.44 N=10.02 (compound D ×H_2_O), found C=69.04 H=11.63 N=10.19; ^1^H-NMR (CDCl_3_, 500 MHz, 27 °C): δ[ppm]= 0.82 [t, ^3^J(H,H) = 6.9 Hz, 6H, 2×-(CH_2_)_2_CH_3_], 1.21-1.79 [m, 65H, -CH_2_-Alkyl, -(CH_2_)_3_CH2NHBOC, -OC(CH_3_)_3_], 1.92-1.99 [m, 8H, 2×-CH_2_CH=CHCH_2_-], 2.39-2.54 [m, 6H, -CH_2_N(CH_2_CH_2_NH_2_)_2_], 2.65-2.71 [m, 4H, 2×-CH_2_NH_2_], 2.99-3.38 [m, 7H, -CH_2_NHBOC, 2×-CH_2_NHCO-, -COCH(oleyl)CO-], 4.29-4.37 [m, 1H, -COCH(NHCO-)CH_2_-], 4.94/5.19 [2×s, 1H, -NHBOC], 5.27-5.32 [m, 4H, 2×-CH=CH-], 7.60/7.91 [2×s, 1H, -COCH(NHCO-)CH_2_-], 6.85/8.18 [2×s, 2H, 2×-NHCO-]

*N*-{6-Amino-1-[*N*-(9*Z*)-octadec-9-enylamino]-1-oxohexan-(2*S*)-2-yl}-*N*`-{2-[*N*,*N*-bis(2-aminoethyl)amino]ethyl}-2-[(9*Z*)-octadec-9-enyl]propandiamide (**OO4**) M(C_51_H_101_N_7_O_3_) = 860.39 g mol^-1^

Yield: 86 %; R_f_: 0.09 (CHCl_3_/methanol/NH_3_, 65/35/5, v/v/v); ESI-MS: 860.6 [M+H]^+^; HRMS calc. 860.8039 Da (C_51_H_102_N_7_O_3_ [M+H]^+^), found 860.8060 Da; ^1^H-NMR (CDCl_3_, 500 MHz, 27 °C) δ[ppm]= 0.85 [t, ^3^J(H,H) = 6.8 Hz, 6H, 2×-CH_3_], 1.24-1.88 [m, 56H, -CH_2_-Alkyl, -(CH_2_)_3_CH_2_NH_2_], 1.93-1.99 [m, 8H, 2×-CH_2_CH=CHCH_2_-], 2.45-2.56 [m, 6H, -CH_2_N(CH_2_CH_2_NH_2_)_2_], 2.64-2.76 [m, 6H, 3×-CH_2_NH_2_], 3.04-3.41 [m, 5H, 2×-CH_2_NHCO-, -COCH(oleyl)CO-], 4.31-4.37 [m, 1H, -COCH(NHCO-)CH_2_-], 5.27-5.35 [m, 4H, 2×‑CH=CH-], 7.16-7.17/7.83-7.98/8.16-8.23 [3×m, 3H; 3×-NHCO-]; ^13^C-NMR (CDCl_3_/CD_3_OD, 125 MHz, 27 °C), δ[ppm]= 12.9, 21.8, 22.11, 22.14, 26.1, 26.3, 28.3, 28.4, 28.50, 28.58, 28.62, 28.7, 28.83, 28.88, 28.92, 28.98, 30.4, 31.0, 31.1, 31.7, 36.6, 36.7, 38.0, 38.1, 38.6, 38.7, 40.1, 40.2, 48.4, 52.69, 52.74, 52.85, 55.26, 55.33, 128.9, 129.0, 129.4, 129.5, 170.4, 170.5, 170.8, 171.5, 171.6

**Supplementary movie 1**

Video for early liposome complexes in NIH3T3 cell, where these complexes exhibited hollowed circular shape with liposomes located along the edge. Most complexes presented brown motion within a restricted area. This video was collected at a rate of 10 frames per second and ran at 4× real time.

**Supplementary movie 2**

Video for time-lapse movie of vesicle-like and tubular structures (yellow) in NIH3T3 cell. The cells were transfected via viral vector transduction (Cell Light Tubulin-GFP) after adherent culture 24 hr. According to our results, this transduction was no obvious effects to the formation and growth of the membrane tubular compartments before, after or simultaneously with liposome incubation. The yellow is a merged color from red liposome complexes and green cytoskeleton MTs. The tubular membrane compartments were definitely produced from liposome complexes, and switched frequently between periods of extension, retraction and oscillation along MTs. The video was collected at a rate of 10 frames per second and ran at 4× real time. **Supplementary movie 3**

Video for time-lapse movie of membrane tubular networks in NIH 3T3 cell. Except Cy5-labeled liposomes invasion, the cell was not applied any other staining processes. Thus, the signal was totally from internalized cationic liposomes. The same field of view was shown in Fig.2D. The video was collected at a rate of 10 frames per second and ran at 4× real time.

**Supplementary movie 4**

Video for “nonstabilized”cytoskeleton MTs without liposome invasion in NIH 3T3 cell. This video was collected at a rate of 10 frames per second and ran at 4× real time.


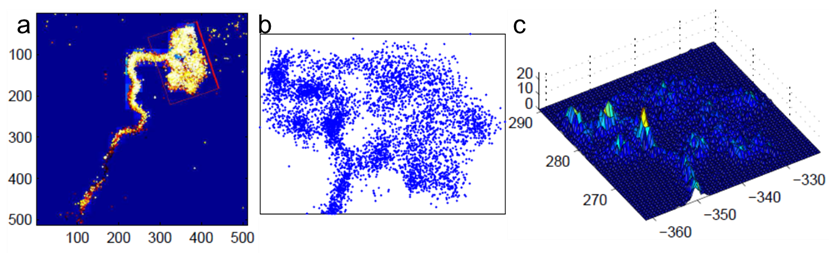


**Supplementary Figure S1.** (a) Detailed dSTORM image of liposome tubular compartments with clusters in NIH 3T3 cells. Both 2D (b) and 3D-view fluorescence signal distributions (c) of the box area suggested liposomes clusters served as a reservoir of linear array or tubules.

**
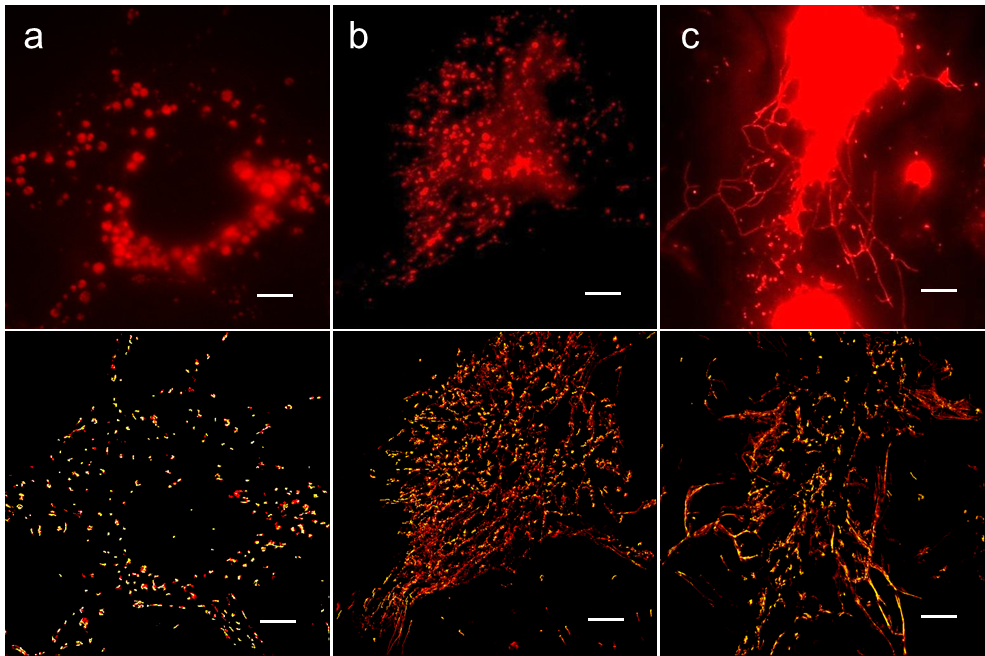
**

**Supplementary Figure S2.** Motility comparison between different stages of liposome complexes in NIH 3T3 cells. The top is one frame of wide field images in a period of 2.5mins (the acquisition speed is 10 frames/second), and the bottom is the corresponding overlay images of trajectories of all moving targets in the view field mapped by 2D Gaussian fitting. The activity and motility keep increasing with time. (a) At early stage (<3h), most liposome complexes remained relatively stationary, and fluctuated in a Brown movement within a restricted area. Only a small percentage of the complex moved actively. (b) At later stage (6-12h), most complexes moved actively with a property of directivity. (c) At reticular stage (>12h, provided plenty of liposome complexes), the active tips of reticular structure move consistently either anterograde or retrograde to form junctions. Scale bar, 5µm


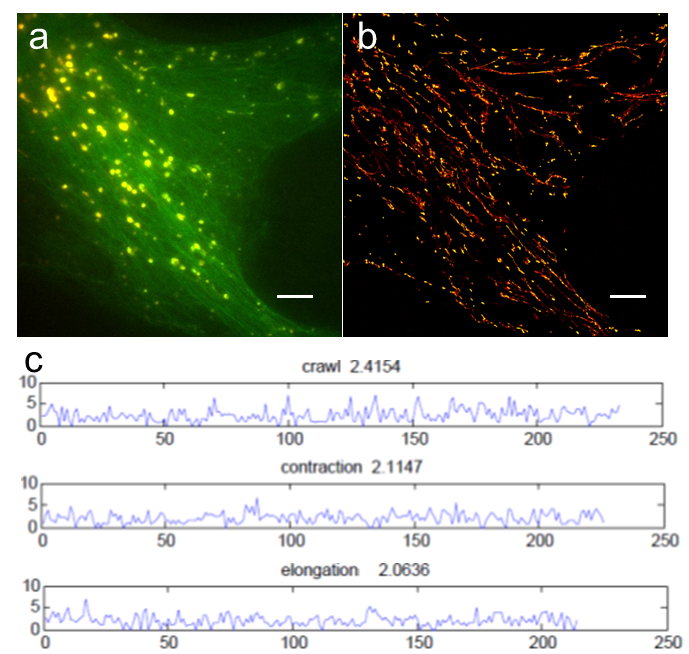


**Supplementary Figure S3.** Motility properties of liposome complexes along cytoskeleton microtubules in NIH3T3 cells. (a) One frame of wide-field images acquired in a period of 2mins (the acquisition speed is 10 frames/sec). (b) An overlay of trajectories of all moving targets in the same view field with (a) mapped by 2D Gaussian fitting. (c) A single liposome complex indicated in (a) was tracked and calculated. Scale bar, 5µm.


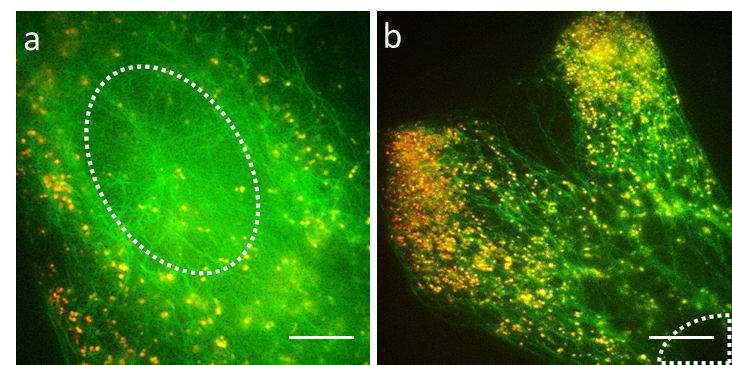


**Supplementary Figure S4. The distribution characters of redispersed liposome compartments via intracellular transporting in living NIH 3T3 cell.** (a) Most vesicular compartments anchored on MTs, and distributed surrounding cell nucleus. (b) In the periphery of the cell, abundant mono-dispersed liposomes located at closets surrounded by MTs. Only the compartments anchored on MTs could be transported actively. Otherwise, they kept at rest or restricted Brown motility. The circled area is cell nucleus. Scale bar, 10µm.


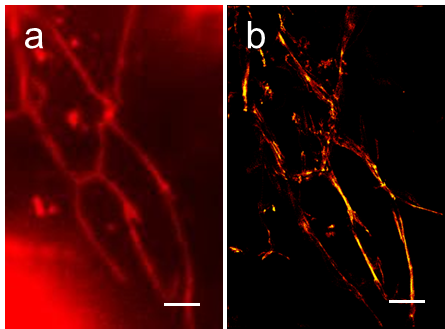


**Supplementary Figure S5.** (a), (b) is wide-field and trajectories overlay image, respectively. The detailed Gaussian fits of selected region from Fig. S2c showed that these reticular structures could keep higher stability, and move consistently either anterograde or retrograde with fewer changes in direction. Scale bar, 2µm.
